# The Vasorelaxant Mechanisms of a Rho Kinase Inhibitor DL0805 in Rat Thoracic Aorta

**DOI:** 10.3390/molecules17055935

**Published:** 2012-05-18

**Authors:** Lili Gong, Jianhao Peng, Lianhua Fang, Ping Xie, Kun Si, Xiaozhen Jiao, Liping Wang, Guanhua Du

**Affiliations:** 1 Beijing City Key Laboratory of Drug Target Identification and Drug Screening, State Key Laboratory for Bioactive Substances and Functions of Natural Medicines, Institute of Materia Medica, Chinese Academy of Medical Science & Peking Union Medical College, Beijing 100050, China; Email: gonglili2010@yahoo.com.cn (L.G.); pengjianhao@hotmail.com(J.P.); xp@imm.ac.cn(P.X.); jiaoxz@imm.ac.cn (X.J.); together3171@hotmail.com(L.W.); 2 Beijing Chao-Yang Hospital affiliated with Beijing Capital Medical University, Beijing 100020, China; 3 Department of Macromolecular Science and Engineering, Case Western Reserve University, Cleveland, OH 44106, USA; Email: kun.si1@case.edu

**Keywords:** DL0805, vasorelaxation, Rho kinase, aorta, endothelium, nitric oxide

## Abstract

Rho-kinase has been suggested as a potential therapeutic target in the treatment of cardiovascular diseases. The Rho-kinase signaling pathway is substantially involved in vascular contraction. The aim of the present study was to evaluate the vasorelaxant effects of Rho kinase inhibitor DL0805 in isolated rat aortic rings and to investigate its possible mechanism(s). It was found that DL0805 exerted vasorelaxation in a dose-dependent manner in NE or KCl-induced sustained contraction and partial loss of the vasorelaxation under endothelium-denuded rings. The DL0805-induced vasorelaxation was significantly reduced by the nitric oxide synthase inhibitor N^ω^-nitro-L-arginine methyl ester, the guanylate cyclase inhibitor methylene blue and the cyclooxygenase inhibitor indomethacin. The voltage-dependent K^+^ channel blocker 4-aminopyridine remarkably attenuated DL0805-induced relaxations. However, the ATP-sensitive K^+^ channel blocker glibenclamide and Ca^2+^-activated K^+^ channel blocker tetraethylammonium did not affect the DL0805-induced relaxation. In the endothelium-denuded rings, DL0805 also reduced NE-induced transient contraction and inhibited contraction induced by increasing external calcium. These findings suggested that DL0805 is a novel vasorelaxant compound associated with inhibition of Rho/ROCK signaling pathway. The NO-cGMP pathway may be involved in the relaxation of DL0805 in endothelium-intact aorta. The vasorelaxant effect of DL0805 is partially mediated by the opening of the voltage-dependent K^+^ channels.

## 1. Introduction

Rho kinase, also known as Rho-associated coiled-coil forming protein kinase (ROCK), is a serine/threonine protein kinase that is the downstream effector of Rho GTPases [1]. The Rho/ROCK pathway is well known as a major regulator of vascular smooth muscle cells (VSMCs) contraction and plays an important role in hypertension, atherosclerosis, and pulmonary hypertension [2,3]. The essential regulatory mechanism of smooth muscle contraction is myosin light chain (MLC) phosphorylation by the Ca^2+^-calmodulin-activated MLC kinase (MLCK) and dephosphorylation by the Ca^2+^-independent MLC phosphatase (MLCP) activity [4]. ROCK mediates the MLC phosphorylation by inhibiting the enzyme activity of MLCP [5,6]. Bussemaker *et al.* demonstrated that ROCK was involved in the regulation of endothelial nitric oxide synthase (NOS) [7,8], while other researchers indicated that NO induces dilation of rat aorta via inhibition of ROCK signaling pathway [9,10]. Rho-kinase inhibitors Y-27632 and fasudil have been used as tool compounds to evaluate the role of ROCK proteins in various disease models. Y-27632 [11] and fasudil [12] have been shown to exhibit relaxation effects on vessels.

5-Nitro-1(2)*H*-indazole-3-carbonitrile (DL0805) is a new ROCK inhibitor derived from a compound database through high-throughput screening (HTS) as previously described [13]. DL0805 is a homologue of 7029, a Rho kinase inhibitor which was found in that previous study [13]. When we evaluated the relaxant effects of the lead compounds identified in the HTS DL0805 showed the strongest vasorelaxant effects. Few studies have been carried out regarding the effect of DL0805 on vascular smooth muscle contraction. Thus, the purpose of the present study was to evaluate the relaxant effects of DL0805 in the rat thoracic aorta and its possible mechanism(s).

## 2. Results and Discussion

### 2.1. Inhibition Effect of DL0805 on ROCK

ROCK-I inhibitory activity was observed in the Kinase-Glo Luminesce Kinase Assay High-throughput screening and virtual screening. As shown in Table 1, the IC_50_ value for DL0805 was determined to be 6.67 μM.

**Table 1:** ROCK-I inhibitors identified by virtual screening and high-throughput screening. Pharmacophore-Fit Value, LigandFit-Dock Score, and IC_50_ values for DL0805 and Y-27632.

Compound ID	Structure	IC_50_ (μM)	FitValue	DockScore
DL0805	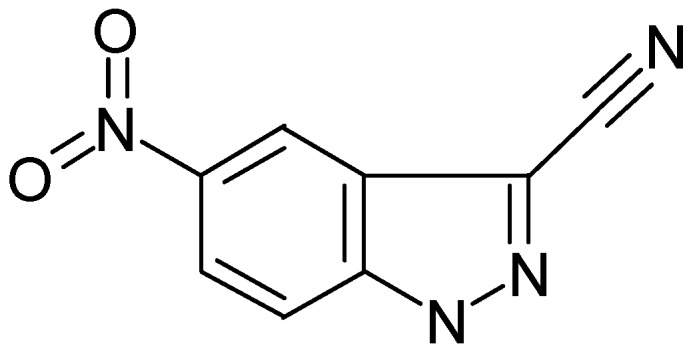	6.67 ± 0.67	1.023	46.25
Y-27632	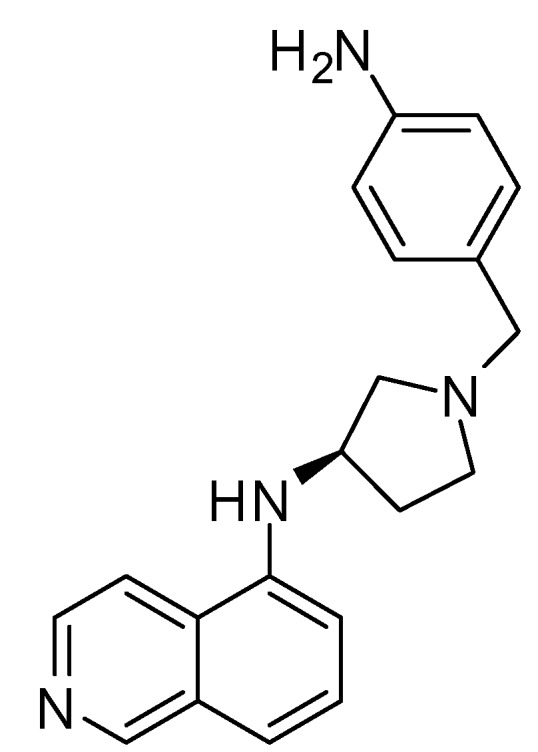	0.20 ± 0.17	3.993	52.49

### 2.2. Relaxant Effects of DL0805 on Aortic Rings Contracted by NE and KCl

**Figure 1:**
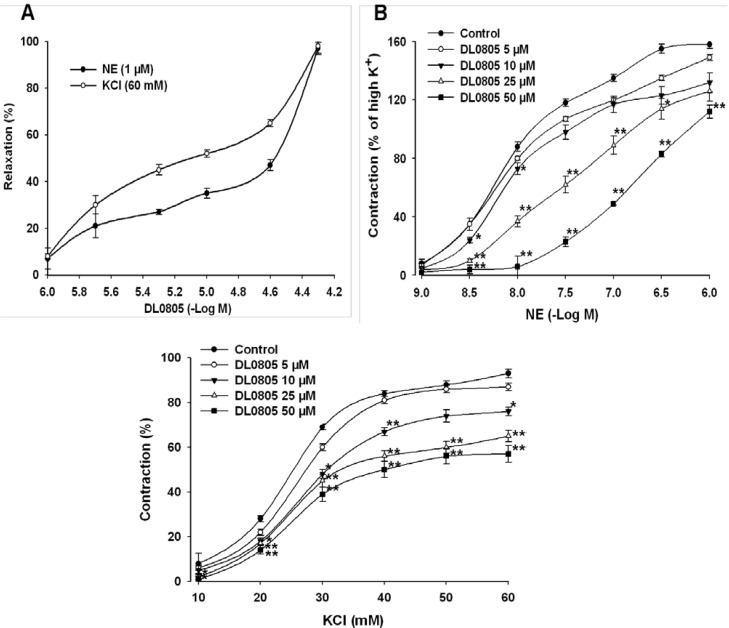
Vasorelaxant effects of DL0805 on endothelium-intact thoracic aorta rings contracted with NE or KCl. (**A**) Effects of DL0805 on endothelium-intact thoracic aorta rings pre-contracted with NE (1 μM) or KCl (60 mM). (**B**) Effects of DL0805 (5, 10, 25, 50 μM) on NE (10^−9^~10^−6^ M) and (**C**) KCl (10~60 mM) induced contraction. Results are presented as means ± S.E.M., n = 6. *P < 0.05, **P < 0.01 compared with vehicle control.

In order to evaluate the effects of DL0805 on the contraction induced by norepinephrine (NE) and KCl, endothelium-intact aortic rings pre-contracted with NE or KCl were used. DL0805 relaxed the NE (1 μM) pre-contracted aortic rings in a dose-dependent manner. DL0805 also relaxed aortic rings pre-contracted with KCl (60 mM) in a similar way (Figure 1A).

Pre-incubation with DL0805 (5, 10, 25 and 50 μM) inhibited the concentration response contraction of NE in a parallel fashion, and depressed the maximal responses to 107.0 ± 4.0%, 101.1 ± 4.5%, 87.3 ± 4.1% and 57.3 ± 2.8%, respectively (*vs.* vehicle control group 111.0 ± 2.0%, n = 6) (Figure 1B) (pA2 value 4.03 ± 0.51; n = 6). We also observed that 5, 10, 25 and 50 μM DL0805 inhibited the contractile response to KCl, and depressed the maximal responses to 87.1 ± 1.7%, 76.5 ± 1.9%, 65.3 ± 2.6% and 57.8 ± 3.7%, respectively (*vs.* vehicle group 93.0 ± 1.9%, n = 6) (Figure 1C) (pIC_50_ value 3.75 ± 0.46; n = 6). 

### 2.3. Role of Endothelium in DL0805 Induced Relaxation of Aortic Ring

**Figure 2:**
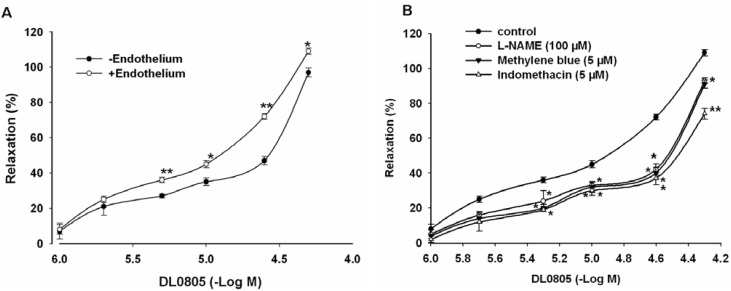
Vasorelaxant effects of DL0805 on the contraction induced by NE (1 μM) in the aortic rings with (+Endo) or without (-Endo) endothelium (**A**) and effects of pre-incubation of L-NAME (100 μM), methylene blue (5 μM) and indomethacin (5 μM) on DL0805 induced relaxation in endothelium-intact aorta (**B**). Results are presented as means ± S.E.M., n = 6. *P < 0.05, **P < 0.01 compared with endothelium-intact aorta (**A**) or *P < 0.05, **P < 0.01 compared with control (**B**).

To elucidate the role of endothelium in DL0805-mediated vasorelaxation, concentration-response to DL0805 was studied in endothelium-intact and endothelium-denuded rings pre-contracted by NE (1 μM). The relaxation effect of DL0805 in endothelium-intact aorta was significantly stronger than that in endothelium-denuded aorta. In endothelium-denuded rings, DL0805 produced a partial relaxation with maximal effect 97.6 ± 2.6% (*vs.* endothelium-intact group 109.1 ± 1.8%, n = 6) (Figure 2A). Removal of functional endothelium inhibited the relaxant response to DL0805, suggesting that the vasorelaxation caused by DL0805 was endothelium-dependent.

Since DL0805 induced both endothelium-dependent and -independent relaxation in isolated rat aortic rings, an attempt was made to investigate what endothelium-derived vasoactive factors contributed to the DL0805-induced relaxation. Pre-incubation of endothelium-intact rings with the NOS inhibitor N^ω^-nitro-L-arginine methyl ester (L-NAME, 100 μM), the guanylate cyclase inhibitor methylene blue (5 μM) and the cyclooxygenase inhibitor indomethacin (5 μM) for 20 min before NE (1 μM) was added, and then DL0805 (1~50 μM) was added. We found that L-NAME, methylene blue and indomethacin significantly reduced the DL0805 induced relaxation, with maximal relaxant effects of 93.3 ± 1.5%, 91.2 ± 2.4% and 74.5 ± 3.2%, respectively (*vs.* control group 109.1 ± 1.8%, n = 6, Figure 2B). These results indicate that the NO-cGMP and endothelium cyclooxygenase pathways may be involved in the relaxation of DL0805 in endothelium-intact aorta.

### 2.4. Role of K^+^ Channels in DL0805 Induced Relaxation

**Figure 3:**
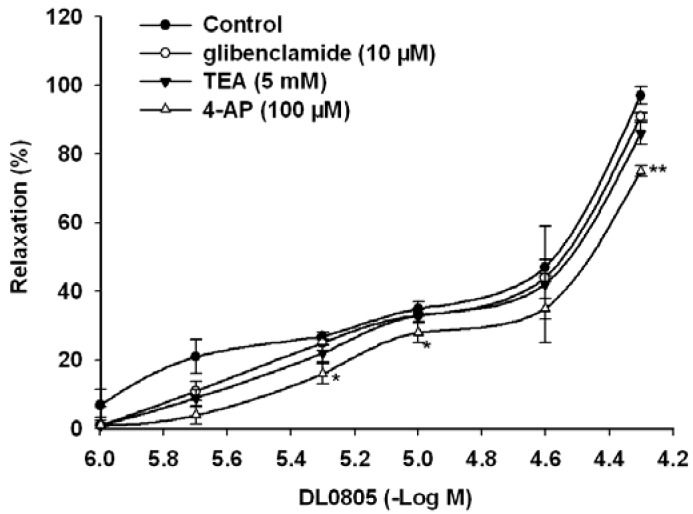
Effects of pre-incubation of glibenclamide (10 μM), TEA (5 mM) and 4-AP (100 μM) on DL0805 induced relaxation in thoracic aorta rings precontracted by NE (1 μM). Results are presented as means ± S.E.M., n = 6, *P < 0.05, **P < 0.01 compared with control.

K^+^ channels play an important role in the regulation of muscle contractility and vascular tone [14]. There are several types of K^+^ conductance present in vascular smooth muscle and they are subject to modulation by various factors. To demonstrate the role of K^+^ channels in DL0805-induced relaxation, endothelium denuded aortic rings were pre-incubated with K^+^ channel blockers. We used three K^+^ channel blockers: the ATP-sensitive K^+^ channel (KATP) blocker glibenclamide, the Ca^2+^-activated K^+^ channel (KCa) blocker tetraethylammonium (TEA), and the voltage-dependent K^+^ channel (Kv) blocker 4-aminopyridine (4-AP). Pretreatment with 4-AP (100 μM) attenuated DL0805-induced relaxations pre-contracted by NE. However, pretreatment with glibenclamide (10 μM) and TEA (5 mM) did not significantly affect the DL0805-induced relaxation (Figure 3). It is probable that DL0805 activates Kv channels in rat endothelium-denuded arteries [15]. These results indicate that the vasorelaxant effect of DL0805 is partially mediated by the opening of the K^+^ channels in VSMCs.

### 2.4. Effect of DL0805 on Extracellular Ca^2+^ Induced Contraction and Intracellular Ca^2+^ Release in Aortic Ring

The influx of external Ca^2+^ through specific Ca^2+^ channels or Ca^2+^ release from internal stores plays an important role in excitation-contraction coupling of smooth muscle. As we know, there are two kinds of Ca^2+^ channels in the VSMCs: Voltage-dependent Ca^2+^ channels and receptor-operated Ca^2+^ channels [16].

**Figure 4:**
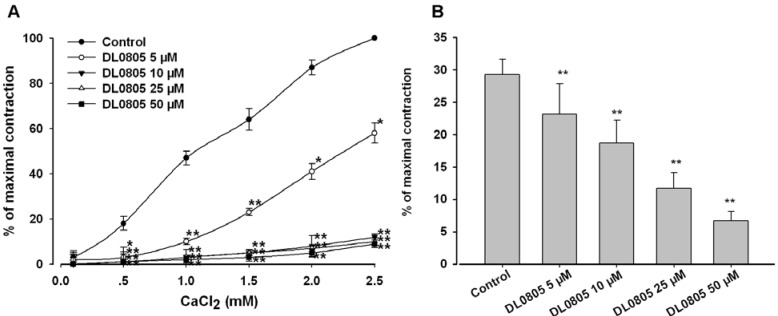
Inhibitory effect of DL0805 on the cumulative-contraction curve dependent on extracellular Ca^2+^ influx induced by KCl (60 mM) in Ca^2+^-free solution added cumulatively CaCl_2_(0.1~2.5 mM) (**A**) and on the NE (1 μM) induced contraction in Ca^2+^-free solution (**B**). Results are presented as means ± S.E.M., n = 6, *P < 0.05, **P < 0.01 compared with vehicle.

To determine whether the inhibition of extracellular Ca^2+^ influx is involved in DL0805 induced relaxation, the experiments were carried out in Ca^2+^-free Krebs-Henseleit (K-H) solution [17]. The high K^+^-induced contraction of smooth muscle is the result of an increase in Ca^2+^ influx through voltage-dependent Ca^2+^ channels [18]. In the Ca^2+^-free solution plus 60 mM KCl, cumulative addition of CaCl_2_ (0.1~2.5 mM) induced a stepwise tension increase of aortic rings. Pretreatment with 5, 10, 25 and 50 μM DL0805 noticeably attenuated CaCl_2_ induced contraction (Emax 58.1 ± 4.4%, 12.7 ± 1.3%, 10.9 ± 1.6% and 9.2 ± 1.7%, respectively, n = 6) (Figure 4A). These results supporting that DL0805 exhibited Ca^2+^ entry blocking activity.

To clarify whether the relaxation induced by DL0805 was related to inhibition of intracellular Ca^2+^ release, experiments were carried out in Ca^2+^-free K-H solution containing 50 μM EGTA [19]. By acting on specific membrane receptors, NE induces Ca^2+^ influx through receptor-operated channels causing tonic contraction [20]. In the Ca^2+^-free solution, NE (1 μM) induced a transient contraction due to the release of intracellular Ca^2+^. As shown in Figure 4B, pretreatment with DL0805 (5, 10, 25 and 50 μM) significantly reduced the contraction induced by NE, and the Emax was 23.1 ± 4.7%, 18.8 ± 3.5%, 11.7 ± 2.5%, 6.6 ± 1.4%, respectively (*vs.* vehicle 29.3 ± 2.4%, n = 6). DL0805 may also inhibit Ca^2+^ mobilization from intracellular stores. Taken together, these results indicate that DL0805 may be acting as a Ca^2+^ antagonist.

## 3. Experimental

### 3.1. Chemicals and Drugs

NE, acetylcholine (ACh), indomethacin, L-NAME, methylene blue, glibenclamide, TEA and 4-AP were purchased from Sigma (St. Louis, MO, USA). DL0805 was synthesized by the Department of Medicinal Chemistry of our institute and its structure was confirmed by the analysis physical-chemical properties and spectral evidence. 

### 3.2. Screening of ROCK InhibitorDL0805

High-throughput screening and virtual screening for the identification of novel ROCK inhibitors was done as previously described [13]. Simply, we developed common-feature pharmacophore models and LigandFit model to compare the structure and the interaction with ROCK between Y-27632 and DL0805. A new high-throughput screening model based on Kinase-Glo Luminescent Kinase Assay (Promega, Madison, WI, USA) was established to identify the inhibition effect of DL0805 on ROCK.

### 3.3. Isometric Tension Measurements

All studies were approved by the Laboratories Institutional Animal Care and Use Committee of the Chinese Academy of Medical Sciences and Peking Union Medical College. Male Sprague-Dawley rats (250–300 g) were anesthetized with pentobarbitone sodium (60 mg/kg, i.p.). The thoracic aorta was immediately excised and immersed in ice-cold K-H solution with the following composition (mM): NaCl 120, KCl 4.8, KH_2_PO_4_ 1.2, NaHCO_3_ 25, glucose 11, CaCl_2_ 2.5, MgCl_2_ 1.4 and ethylene-diaminetetraacetic acid (EDTA) 0.01. After removed debris tissue, the aorta was cut into rings of about 2~3 mm in length. For endothelium-denuded aorta, endothelium was mechanically removed by gently rubbing the lumen with a wet cotton ball and the absence of acetylcholine-induced relaxation was used as a denuding indicator [21].

The tension of aortic rings was recorded isometrically via a force displacement transducer connected to a BIO-PAC polygraph (MP100A). The aortic rings were hanged in organ baths containing 10 mL K-H solution which was maintained at 37 °C and gassed continuously with a 95% O_2_ and 5% CO_2_ mixture [22] and equilibrated for 60 min under a 1.2 g resting tension. During the equilibration period, K-H solution was changed every 20 min. The aortic rings were given two successive stimulations with high K^+^ (60 mM) solution, which was prepared by replacing NaCl with equimolar KCl in K-H solution. The tension reached about 3 g when treated with 60 mM K^+^. The endothelial integrity was confirmed by eliciting a relaxation with ACh (10 μM) after contraction induced by NE (1 μM). Only endothelium-intact rings exhibiting more than 60% relaxation to ACh were used for the experiments [23]. While in endothelium denuded rings, the relaxation to ACh was less than 5%. Submaximal contraction was induced using NE (1 μM), then cumulative dose-response curves to the DL0805 (1~50 μM). With inhibitors (L-NAME, methylene blue or indomethacin) or vehicle, the vessels were pretreated with such compounds prior to submaximal contraction with NE then determined the endothelial response to the tested compounds. TEA, glibenclamide and 4-AP were also used to demonstrate the role of K^+^ channels on DL0805 induced relaxation. In order to clarify the Ca^2+^ release, the experiments were carried out in Ca^2+^-free K-H solution.

### 3.4. Statistical Analysis

All data were expressed as the means ± S.E.M. Statistical analysis was performed using the one-way ANOVA or Student’s t-test. The *P* value less than 0.05 was regarded as significantly different.

## 4. Conclusions

In conclusion, the results suggest that DL0805 exerts its vasodilatory effects by acting on multiple sites. DL0805 induces relaxation in rat aortic rings through endothelium-dependent and -independent pathways. NO-cGMP mediated pathway may be involved in the endothelium-dependent relaxation. DL0805 also blocks extracellular Ca^2+^ influx by interacting with both voltage- and receptor-operated Ca^2+^ channels. The Rho/ROCK pathway has been shown to induce vascular smooth muscle contraction, suppress the expression of eNOS, and increase intracellular Ca^2+^ concentration. DL0805 act as a novel vasorelaxant compound associated with inhibition of Rho/Rho-kinase signaling pathway.
